# Genome-Wide Mapping of Quantitative Trait Loci for Yield-Attributing Traits of Peanut

**DOI:** 10.3390/genes15020140

**Published:** 2024-01-23

**Authors:** Pushpesh Joshi, Pooja Soni, Vinay Sharma, Surendra S. Manohar, Sampath Kumar, Shailendra Sharma, Janila Pasupuleti, Vincent Vadez, Rajeev K. Varshney, Manish K. Pandey, Naveen Puppala

**Affiliations:** 1 International Crops Research Institute for the Semi-Arid Tropics (ICRISAT), Hyderabad 502324, India; pushpeshjoshi92@gmail.com (P.J.); vinaysharma965@gmail.com (V.S.); m.surendra@cgiar.org (S.S.M.); janila.pasupuleti@icrisat.org (J.P.); rajeev.varshney@murdoch.edu.au (R.K.V.); 2 Department of Genetics and Plant Breeding, Chaudhary Charan Singh University, Meerut 250004, India; shgjus6@gmail.com; 3 Agricultural Research Station, Andhra Pradesh Agricultural University, Anantapur 515591, India; dsampath2020@gmail.com; 4 Institut de Recherche pour le Development (IRD), Université de Montpellier, Unité Mixte de Recherche Diversité et Adaptation des Espèces (UMR DIADE), 34394 Montpellier, France; vincent.vadez@ird.fr; 5 Centre for Crop and Food Innovation, Food Futures Institute, Murdoch University, Murdoch, WA 6150, Australia; 6 Agricultural Science Center at Clovis, New Mexico State University, Clovis, NM 88101, USA

**Keywords:** yield, phenotype, genes, markers, epistatic

## Abstract

Peanuts (*Arachis hypogaea* L.) are important high-protein and oil-containing legume crops adapted to arid to semi-arid regions. The yield and quality of peanuts are complex quantitative traits that show high environmental influence. In this study, a recombinant inbred line population (RIL) (Valencia-C × JUG-03) was developed and phenotyped for nine traits under two environments. A genetic map was constructed using 1323 SNP markers spanning a map distance of 2003.13 cM. Quantitative trait loci (QTL) analysis using this genetic map and phenotyping data identified seventeen QTLs for nine traits. Intriguingly, a total of four QTLs, two each for 100-seed weight (HSW) and shelling percentage (SP), showed major and consistent effects, explaining 10.98% to 14.65% phenotypic variation. The major QTLs for HSW and SP harbored genes associated with seed and pod development such as the *seed maturation protein-encoding* gene, *serine-threonine phosphatase* gene, *TIR-NBS-LRR* gene, *protein kinase superfamily* gene, *bHLH transcription factor*-encoding gene, *isopentyl transferase* gene, *ethylene-responsive transcription factor*-encoding gene and *cytochrome P450* superfamily gene. Additionally, the identification of 76 major epistatic QTLs, with PVE ranging from 11.63% to 72.61%, highlighted their significant role in determining the yield- and quality-related traits. The significant G × E interaction revealed the existence of the major role of the environment in determining the phenotype of yield-attributing traits. Notably, the *seed maturation protein-coding* gene in the vicinity of major QTLs for HSW can be further investigated to develop a diagnostic marker for HSW in peanut breeding. This study provides understanding of the genetic factor governing peanut traits and valuable insights for future breeding efforts aimed at improving yield and quality.

## 1. Introduction

Peanuts (*A. hypogaea* L.) hold a prominent position among oilseed crops due to their numerous nutritional attributes. They are widely recognized as an excellent source of edible oil, providing a high-quality oil that is widely used in cooking and food preparation. In addition to their oil content, peanuts are also valued for their protein content. Furthermore, peanuts offer dietary fiber, which aids in proper digestion and therefore is considered crucial for digestive health. Peanuts pack a range of essential minerals and vitamins, including magnesium, phosphorus, potassium, and B vitamins like niacin and folate. These nutrients play vital roles in various bodily functions, such as energy production, bone health, and nerve function [1]. Their shells are used in the animal feed, fuel, and fertilizer industries [2]. The haulm, typically utilized as animal feed, serves the dual purpose of providing fodder while also contributing to nitrogen fixation in the soil. The nitrogen fixation capability of the haulm can range from 100 kg ha^−1^ to 152 kg ha^−1^ [3]. Peanuts are grown in more than 100 countries worldwide, producing 53.9 million metric tons (mt) from 32.7 million ha area [4]. The crop is grown commercially between 40° N and 40° S latitudes; the largest producer in the world is China (17.9 mt), followed by India (9.9 mt) and Nigeria (4.5 mt). Peanut production is confronting severe biotic and abiotic stresses due to climate change, which emphasizes the necessity of climate-resilient crop production considering global food security. Agronomic traits such as biomass, pod weight, seed weight, and shelling percentage largely influence the yield and play a major role in the domestication, breeding, and selection of new peanut cultivars [5]. Cultivated peanut around the world has a narrow genetic base. The linkage drag of desirable and undesirable traits often imposes biological constraints in developing improved cultivars by conventional crossing and selection [6]. The crucial factors that affect the yield are the 100-pod weight (HPW), 100-seed weight (HSW), haulm yield, and shelling percentage (SP) [7]. Traditional breeding methods face challenges when dealing with these quantitative traits. Moreover, these traits are typically governed by several genes, each with a relatively modest impact, which adds to their complexity and makes the breeding process laborious and time-consuming. Utilizing genomics-assisted breeding (GAB) plays a vital role in enhancing peanut yields substantially. Through the application of GAB, researchers can pinpoint and choose particular genes linked to yield-attributing traits, facilitating the more efficient development of high-yielding varieties compared to relying solely on traditional breeding methods [8].

Furthermore, genotype by environment interaction (G × E) plays a significant role in determining the cultivar performance, imposing constraints in identifying the traits needed to improve productivity. The last decade has witnessed an increased demand for peanuts compared to other oilseed crops due to the increased use of peanuts in confectionary, health-sensitive consumers, and benefits to traders. Notably, the phenotypic selection of lines having significantly higher seed weight is difficult in standing crops. The availability of linked markers to seed weight will unfurl the scope of marker-based early-generation selection. The sequenced diploid ancestors have provided insights into understanding the genome of cultivated type [9]. Evolutionary studies report low levels of genetic variation and polymorphism between the two sub-genomes [10,11]. 

Over the past five years, the increasing availability of genomic resources for wild species in peanut has opened up new avenues for the exploitation of genetic potential [12]. Several studies were carried out to construct a genetic map to identify quantitative trait loci (QTL) associated with yield and its attributing traits [13,14,15]. To date, a few SSR-based genetic maps are available for peanuts [16,17,18]. However, the use of SSR markers is time-consuming and labor-intensive. It has low throughput [19], while the presence of abundant genome-wide single-nucleotide polymorphisms (SNPs) can be exploited for map construction and for identifying genomic regions that control target traits [20]. Various approaches, including genome-wide association studies, GWASs [21], bulked segregant analyses, BSAs [16], and specific-locus amplified fragment sequencing, SLAF-seq [22] have been experimented upon for the identification and narrowing of the genomic regions/QTLs associated with yield- and quality-related traits in peanuts [5,23]. Recently, it has been shown that the QTL-seq approach could help in identifying a 1.89 Mb region on chromosome B06 linked to seed weight [24] and overlapped regions on A09 and B02 for shelling percentage [25]. Similarly, the identification of 36 marker–trait associations (MTAs) for pod length, pod length–width ratio, and 100-pod weight [21] and six QTLs for seed weight [23] added significantly to the understanding of the genetic basis of these traits. A total of three overlapping QTL hotspots were identified for haulm weight, pod weight, 100-seed weight, and SP, indicating the significant impact of these traits on peanut yield [26].

Additionally, mapping minor alleles and their interactions is key to understanding their role in genomic-assisted breeding for improving yield-related traits [27]. Most studies have targeted only the additive effects of genetic components, whereas minor alleles and epistatic interactions have remained unaddressed. Apart from additive QTLs, the phenotype of a plant is also regulated by epistatic QTLs and, therefore, should be considered in QTL analysis studies [28,29]. The intricate polygenic nature of the yield-attributing traits, its low heritability, minor allele interactions, and the substantial G × E interactions pose limitations on developing high-yielding cultivars that can perform well across diverse locations [30]. Therefore, the objective of the current study was to use a recombinant inbred line (RIL) population (Valencia-C × JUG-03) to address the minor alleles’ interactions and identify the genomic regions and candidate genes associated with yield- and quality-related traits. GBS-based genotyping data were used to construct a dense genetic map. The genetic map, genotyping data, and multi-environment phenotyping data were used to identify the genomic regions associated with yield- and quality-related traits in peanuts.

## 2. Materials and Methods

### 2.1. Phenotyping RIL Population for Yield and Quality Traits

An RIL population comprising 288 lines derived from a cross between Valencia-C × JUG 03 was developed and advanced by a single-seed descent method [31,32]. ‘New Mexico Valencia-C’ is a drought-susceptible and high-yielding Valencia-type cultivar with a higher percentage of 3–4 seeded pods and relatively early yielding compared to other Valencia cultivars [33]. JUG 03 is a drought-tolerant, relatively low-yielding cultivar derived from the cross ICGS 76 × CSMG 84-1 released in Gujarat, India. RILs were phenotypically assessed for yield and quality traits across two environments in India, ICRISAT-Patancheru (E1) (17.51° N, 78.27° E, 920 m), and Kadiri (E2) (14.11° N, 78.16° E, 2572 m) during the rainy season of 2019, with two replications in randomized block design. The plants were planted in the field with a spacing of 30 × 10 cm. All recommended agronomical practices were followed when conducting experimental procedures at each location.

#### 2.1.1. Phenotyping for Yield-Related Traits

The RIL population was evaluated across two locations for yield-related traits, including pod yield, haulm yields, HPW, HSW, and SP. The pod yield was calculated by multiplying the pod weight per plant and the number of plants in one ha area and measured in kg ha^−1^ [34]. Similarly, haulm yield was calculated by multiplying the haulm weight per plant and the number of plants in one ha area and measured in kg ha^−1^. HSW was measured as the weight of one hundred seeds in grams (g), and HPW was calculated as the weight of one hundred pods in g. Shelling percentage was measured as the percentage of the ratio of kernel weight to the total pod weight.

#### 2.1.2. Phenotyping for Physiological Traits

A soil plant analytical development (SPAD) chlorophyll meter provides a dimensionless measurement which is determined by comparing the amount of light absorbed at 430 nm (the optimal wavelength for chlorophyll a and b) with the amount absorbed at 750 nm (near-infrared) with no transmittance. As a result, the readings obtained from an SPAD chlorophyll meter (SCMR) indicate the concentration of chlorophyll in a leaf. The SCMR was measured using Minolta SPAD 502 (Tokyo, Japan) from the third completely expanded leaf from the top of the main stem as per the procedure described by [35]. 

#### 2.1.3. Phenotyping for Oil Content, Fatty Acids, and Protein Content

The oil content, protein estimation, and fatty acids such as linoleic acid and oleic acid were phenotypically assessed in a population of 288 RIL individuals using near-infrared reflectance spectroscopy (NIRS) (Model XDS RCA, FOSS Analytical AB, Sweden, Denmark) and measured in percentages [36].

### 2.2. Phenotypic Analysis

The analysis of phenotypic data was carried out by using the packages “doebioresearch version 0.1.0” and “Variability version 0.1.0” in R software [37]. Analysis of variance (ANOVA), estimation of phenotypic and genotypic coefficient of variation (PCV and GCV), and broad sense heritability values were found for both environments. 

### 2.3. DNA Isolation and Sequencing

The leaf samples were taken from the plants 25–30 days after being sown from the RIL population and two parents for DNA isolation. The Nucleospin Plant II kit (Macherey-Nagel, Düren, Germany) was used for isolating the DNA from the collected leaf samples [38]. The DNA quality was checked on 0.8% agarose gel. Genotyping-by-sequencing (GBS) [39] was performed for the 288 RILs to identify the SNP. For this, 10 ng of DNA from each line was digested using restriction endonuclease *ApeKI.* This enzyme recognizes the site G/CWCG followed by the ligation of barcode adapters to digested products. An equal proportion of adapter-ligated fragments was used for library construction. These libraries were filtered by amplifying to remove additional adapters. These libraries were sequenced on the HiSeq 2500 platform (Illumina Inc., San Diego, CA, USA) to produce numbers of sequence reads. 

### 2.4. SNP Calling and Filtering

TASSEL v4.0 [40] was used for SNP discovery from the FASTAQ files of raw sequence reads of the RILs and parents. For SNP calling, the draft genome sequences of diploid progenitors (*A. ipaensis* and *A. duranensis*) were used as the reference genome assembly [9]. Perfectly matched barcodes with four base remnants of the digestion site of the restriction enzyme in sequencing reads generated for RIL and parental genotypes were detected using in-house script. The sorting and de-multiplexing of the sequence reads was carried out using the above information on barcoding. Trimming was carried out on available reads up to the first 64 bases, starting from the restriction site of the endonuclease enzyme. The reads containing ‘N’ within the first 64 bases were identified and filtered. The remaining sequence reads (tags) were aligned on draft genome sequences of progenitors using the Burrows–Wheeler Alignment (BWA) tool [41]. None of the lines had >50% missing information and ≤0.3 minor allele frequency (MAF). The FSFHap algorithm was executed in TASSEL v4.0 throughout the mapping population to identify and impute missing data. Further, the filtrations of MAF with a yardstick of 0.2 were applied to remove missing SNPs, and the resultant SNPs were used for genetic mapping and QTL analysis [42]. 

### 2.5. Construction of Genetic Linkage Map and QTL Analysis

Usually, the markers had a segregation ratio of 1:1 for the RIL population. Those SNPs were said to be distorted, which were not as per the expected segregation ratio. The Chi-square (χ^2^) values calculated for each SNP marker were used to determine the goodness of fit to the expected 1:1 segregation ratio; highly distorted and unlinked markers were filtered out and not considered for the linkage map construction The linkage map was constructed using JoinMap v4 [43]. The regression mapping algorithm was used for the grouping and ordering of markers. The recombination frequency was converted into map distance (cM) using Kosambi’s mapping function. Those markers that had zero cM intervals had zero recombination frequency. Based on LOD scores ranging from 3 to 10 and a minimum recombination frequency threshold of 50%, the markers were arranged in an orderly manner in 20 linkage groups (LGs). The package “LinkageMapView” version 2.1.2 was used to draw final linkage map in R-studio [44]. The genotypic and phenotypic data, along with the linkage map, were used for composite interval mapping (CIM) in QTL Cartographer Version 2.5_011 [45]. The presence of a QTL was declared based on the threshold LOD determined by permutation at 1000 times for each trait separately. LOD threshold value ≥3 and PVE >10% were considered as major QTLs.

### 2.6. Identification of Candidate Genes from Identified QTL Regions and Expression Analysis

The genomic region of a marker located in close proximity to the identified QTL peak spanning 2 Mb upstream and downstream was used to study the candidate genes in peanut base (https://Peanutbase.org/ accessed on 24 December 2021) through GBrowse (*A. duranensis* and *A. ipaensis*) aradu.V14167.gnm1.ann1.cxSM version 1 and araip.K30076.gnm1.ann1.J37 m version 1. The investigation of tissue-specific expression patterns of the identified candidate genes was conducted using the *A. hypogaea* gene expression atlas (AhGEA) specific to the *fastigiata* sub-species (BioProject ID: PRJNA484860) [46].

### 2.7. Identification of Epistatic (Q × Q) Effect 

It is well established that the inheritance of yield- and quality-related traits is complex; therefore, epistatic QTLs were also evaluated for identifying the effect of two or more genomic regions on the trait expression using inclusive composite interval mapping for epistatic (ICIM-EPI) with a step of 5 cM and 0.001 probability in ICIMapping version 4.1 [47]. The LOD threshold was determined by permutation at 1000 times as the minimum significance level for epistatic QTLs for each trait separately.

## 3. Results

### 3.1. Phenotypic Data Analysis

ANOVA revealed significant variation among RILs for all the traits in both environments, except for haulm yield and oleic acid in Kadiri (E2) (Supplementary Table S1). Pooled ANOVA revealed highly significant G × E interactions for pod yield, HPW, HSW, SP, and SCMR. However, significant variation between environments was found only for pod and haulm yields. Among genotypes (G), significant variation was found for haulm yield, oil content, protein content, linoleic acid, and oleic acid (Table 1) (Supplementary Table S2).

Among RILs, Patancheru (E1) has a broader range for traits, HPW, HSW, and SCMR compared to E2, whereas a narrow range was observed in E1 for the traits of pod yield, haulm yield, SP, oil content, linoleic acid, and oleic acid when compared to E2. In the comparison between E1 and E2, the pod yield, HSW, SCMR, protein content, and linoleic acid levels showed numerically higher values in E1 for both parents and RILs, but these differences were not statistically significant. However, the mean of haulm yield was higher at E2 for both parents and RILs (Table 2).

**Figure 1:**
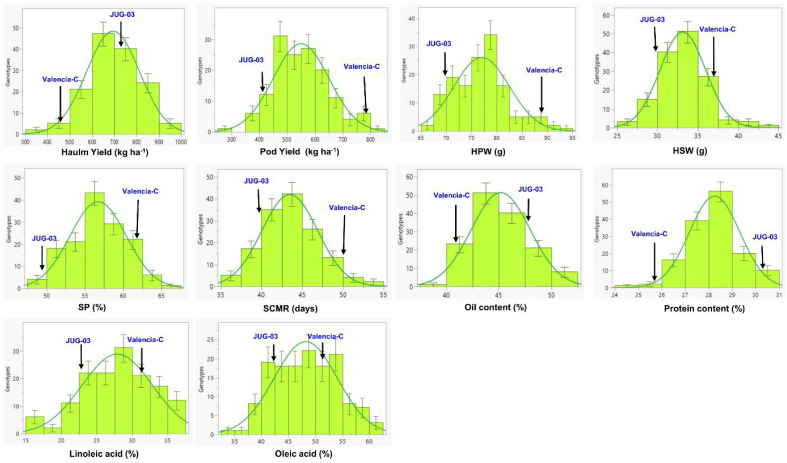
The histogram showing normal distribution for all the traits in the RIL (Valencia-C × JUG-03) population pooled over two environments. HPW: 100-pod weight (g); HSW: 100-seed weight (g); SP: shelling percentage; SCMR: SPAD chlorophyll meter reading.

The parent, Valencia-C, had significantly higher pod yield, HPW, and SCMR than JUG-03 in both environments. However, Valencia-C showed a marginal advantage over JUG-03 in terms of HSW, SP, linoleic acid, and oleic acid. It is important to note that these differences were not statistically significant, indicating that the observed superiority of Valencia-C in these traits may be minimal or due to random variation. As compared to Valencia-C, JUG-03 was significantly higher in magnitude for protein content only. However, when considering haulm yield and oil content, JUG-03 only displayed a numerical superiority over Valencia-C without reaching statistical significance. None of the traits had high heritability (>60%) in either environment. The frequency distribution of pooled data for all the traits revealed a normal distribution, showing their inheritance’s quantitative nature. For all the traits, the majority of the RILs were within the parental limit, and few transgressive segregants were observed in either direction (Figure 1).

### 3.2. Identification of Marker Polymorphism and Genotyping 

A total of 78,614 SNPs on 288 RILs were generated to construct the linkage map. SNPs carrying more than 80 percent missing information were filtered out. Further, a total of 33,245 SNPs were filtered to remove monomorphic and heterozygous loci. Thereby, distorted markers were removed using chi-square to end the disturbance produced during linkage map construction. Therefore, a total of 3393 polymorphic SNPs remained and were used for the construction of the linkage map.

### 3.3. Construction of Genetic Linkage Map

A total of 1323 loci were mapped on 20 linkage groups, spanning 2003.13 cM. In total, 558 SNP loci were mapped in the A-subgenome spanning 1079.47 cM, whereas 749 SNP loci in B-subgenome covered 923.66 cM distance. The number of mapped loci among chromosomes varied from 20 (B09) to 124 (B05), with map length ranging from 55.0 cM (A07) to 167.1 cM (A08), and the average inter-marker distance ranged from 0.60 cM (B05) to 3.71 cM (A08) (Table 3).

### 3.4. QTLs for Yield and Quality Traits

**Figure 2:**
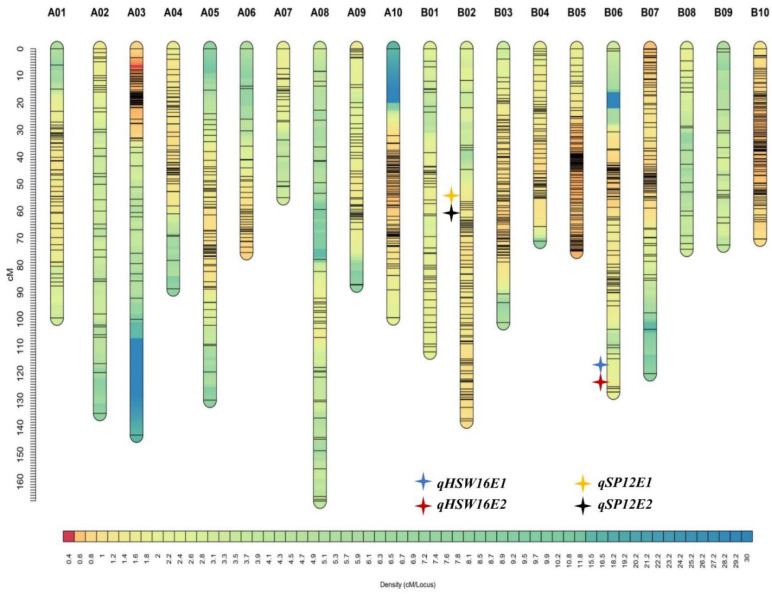
A genetic map of Valencia-C × JUG-03 mapping population showing major QTLs for HSW and SP, which are shown on the left side of the corresponding linkage group.

Phenotypic and genotypic information on yield- and quality-related traits were used to identify QTLs across the two environments. The threshold LOD was estimated for each trait separately based on permutation run 1000 times (Supplementary Table S3). A total of 17 QTLs for nine traits were detected, with the PVE ranging from 4.46% (oil content) to 14.65% (HSW). Of these seventeen QTLs, three QTLs were identified for pod yield, two for haulm yield, one for SCMR, three for HSW, two for SP, two for oleic acid, two for oil, and one for protein and one for linoleic acid. Four major QTLs, two for HSW and two for SP, are mapped on chromosomes B06 and B02, respectively. (Table 4) (Figure 2).

#### 3.4.1. QTLs for Yield-Related and Physiological Traits

For pod yield, a QTL (*qPODYLD18E1*) with LOD- 4.88 and PVE of 6.87% was detected on the B08 chromosome under the E1 environment, whereas two QTLs, namely *qPODYLD12.2E2* (LOD- 4.6 and PVE- 6.27%) and *qPODYLD12.3E2* (LOD- 4.78 and PVE- 6.53%), were found on the B02 chromosome in the E2 environment. A total of two QTLs, named *qHAULMYLD18E1* (LOD- 4.34 and PVE- 6.09%) and *qHAULMYLD6E1* (LOD- 3.64 and PVE- 9.55%), were detected on chromosome B08 and A06, respectively, in E1 for haulm yield. Two QTLs, named *qHSW12E1* (LOD- 4.06 and PVE- 5.89%) and *qHSW16E1* (LOD-6.65 and PVE- 14.65%), for HSW, were located on chromosome B02 and B06, respectively, in E1. However, only a single QTL, *qHSW16E2* (LOD- 6.11 and PVE- 13.87%), was found in E2 on chromosome B06. The genomic region of S16_2332048-S16_8231918 harbored two consistent QTLs (*qHSW16E1* and *qHSW16E2*) with favorable alleles contributed by Valencia-C. A QTL, *qSP12E1* (LOD- 4.36 and PVE- 10.98%), was found on chromosome B02 in E1, and another QTL, *qSP12E2* (LOD- 4.43 and PVE- 11.65%) was found on chromosome B02 in E2. These two QTLs were found in the same marker interval (S12_42838843-S12_73270208) of chromosome B02 with favorable alleles contributed by JUG-03. For SCMR, a single QTL, *qSCMR17E2* (LOD- 4.27 and PVE- 5.90%), was detected on chromosome B07 in E2.

#### 3.4.2. QTLs for Oil Content, Fatty Acids, and Protein Content

The two QTLs, *qOIL12E1* (LOD- 3.05 and PVE- 4.49%) and *qOIL12E2* (LOD- 3.02 and PVE- 4.46%), were found within the same genomic region (S12_42838843-S12_73270208) of chromosome B02 in E1 and E2, respectively. A QTL, *qPROTEIN12E2* (LOD- 3.15 and PVE- 4.66%), was detected within the genomic region, S12_42838843-S12_73270208, located on chromosome B02 in E2. For oleic acid, a QTL named *qOLEIC17E1* (LOD- 5.76 and PVE- 8.09%) was found on chromosome B07 in E1, whereas another QTL, *qOLEIC17E2* (LOD-6.14 and PVE- 8.50%), was detected on chromosome B07 in E2. However, for linoleic acid, a single QTL, *qLINOLEIC12E2* (LOD- 3.43 and PVE- 5.15%), was found in the marker interval of S12_36421620-S12_118023921, located on chromosome B02 in E2 (Table 4).

### 3.5. Epistatic (QTL × QTL) Interaction for Yield- and Quality-Related Traits

A summary of epistatic interactions is provided in Table 5**,** and pairwise detailed information is presented in Supplementary Table S4. A total of 77 epistatic QTLs with PVE between 9.31and 72.61% were detected for nine traits. Among these 77, 76 epistatic QTLs had PVEs of more than 10%, indicating their major effect on the traits.

#### 3.5.1. Digenic Interaction

**Figure 3:**
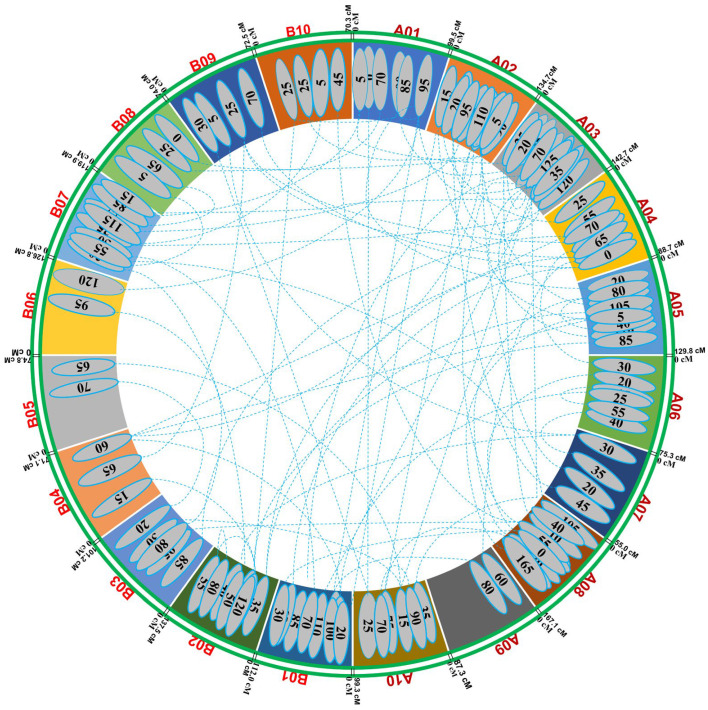
Representation of epistatic QTLs for yield- and quality-related traits in Valencia-C × JUG-03 RIL population.

For pod yield, nine pairs of epistatic interactions with significant additive effects (*p* ≤ 0.0001) involving 13 loci mapped on A01, A03, A04, A05, A07, B01, B02, B03, B04, and B09 were detected—the epistatic interaction, 3–130/5–25, produced an effect larger than other significant interactions. Most of the digenic interactions were between minor-effect QTLs. For haulm yield, 15 pairs of epistatic interactions with significant additive effects (*p* ≤ 0.0001) involving 27 loci distributed in chromosomes A02, A03, A04, A07, A08, A10, B01, B02, B07, B04, and B08 were detected. The epistatic interaction, 17–85/18–65, had the largest effect of all interactions. Four pairs of epistatic interactions were identified for HSW that involved eight loci in chromosomes A02, A05, A08, B03, B06, and B10. All the pairs showed significant additive effects, which indicated that the combined effect of specific genetic loci on different chromosomes influenced the HSW more significantly than the individual effects of each locus alone. However, pair 2–5/5–35 had the largest additive effects. This indicated that when the alleles at locus 5 cM on chromosome A02 interacted with the alleles at locus 35 cM on chromosome A05, there was a substantial increase in the HSW. This specific epistatic interaction played a crucial role in determining HSW, highlighting the importance of these genetic regions in influencing the HSW. For SCMR, eight pairs of interactions were found with 14 significant additive effects, showing loci in the chromosomes A03, A04, A05, A06, A08, B01, B02, B03, B04, and B09. For SP, 14 significant interaction pairs were detected, involving 25 loci mapped on all the chromosomes except A01, B04, B05, B06, B08, and B10. All the loci had significant additive effects at the 0.0001 probability level. The digenic interaction 3–20/3–125 had the largest effect of all interactions. Seven pairs of digenic interactions that had significant effects were detected for oil content, including 12 loci dispersed on chromosomes A01, A02, A03, A08, A10, B02, B07, B08, and B10. All the loci showed significant additive effects (*p* < 0.0001), and only the parental genotype had positive effects for all the pairs of interactions. Eleven epistatic interactions were identified for protein content, including 19 loci located on all chromosomes except A03, A05, A08, B02, and B09. All the loci had significant additive effects; however, the 9–80/11–100 pair showed the largest additive effect (Figure 3). 

#### 3.5.2. Trigenic Interaction 

For haulm yield, the QTL chromosomal interval 3–130 on chromosome A03 interacted with two different loci, 17–15 on chromosome B07 with PVE- of 51.05% and 8–105 on chromosome A08 with PVE- of 43.05%, showing the trigenic interaction. The parent Valencia-C had a positive effect on the haulm yield. Moreover, the genomic region 3–130 interacted with locus 5–25, had PVE- of 38.48%, and influenced the pod yield. For pod yield, three trigenic interactions were observed. The locus 1–95 located on chromosome A01 interacted with loci 3–115 on chromosome A03 with PVE- of 34.09% and 5–20 on chromosome A05 with PVE- of 25.47%. Similarly, another locus 4–55 on chromosome A04 influenced two loci, 13–85 on chromosome B03 with PVE- of 28.38% and 14–60 on chromosome B04 with PVE- of 24.96%. The locus 7–30 on chromosome A07 interacted with loci 12–40 on chromosome B02 with PVE- of 27.23% and 19–5 on chromosome B09 with PVE- of 25.16%. In all three cases, favorable unfavorable alleles were contributed by JUG-03. However, locus 1–95 also interacted with two other loci 11–90 on chromosome B01, showing PVE- of 61.59%, and 16–120 on chromosome B06 with PVE- of 61.22%, which influenced protein content. The parent Valencia-C had a positive effect on protein content. Other locus-governing protein content was 2–90 on chromosome A02 which interacted with two loci, 14–60 with PVE- of 60.97% and 7–20 with PVE- of 50.04% on chromosome A07. The locus 1–5 on chromosome A01 influenced the loci 17–15 with PVE-28.23% on chromosome B07 and 3–90 with PVE- of 21.85% on chromosome A03 for oil content. For SP, locus 3–125 on chromosome A03 interacted with two loci, 8–25, showing PVE- of 72.61% on chromosome A08 and 10–15 on chromosome A10 with PVE- of 69.46%. Moreover, the favorable allele was contributed from Valencia-C (Supplementary Table S4).

### 3.6. Identification of Candidate Genomic Regions for HSW and SP

**Figure 4:**
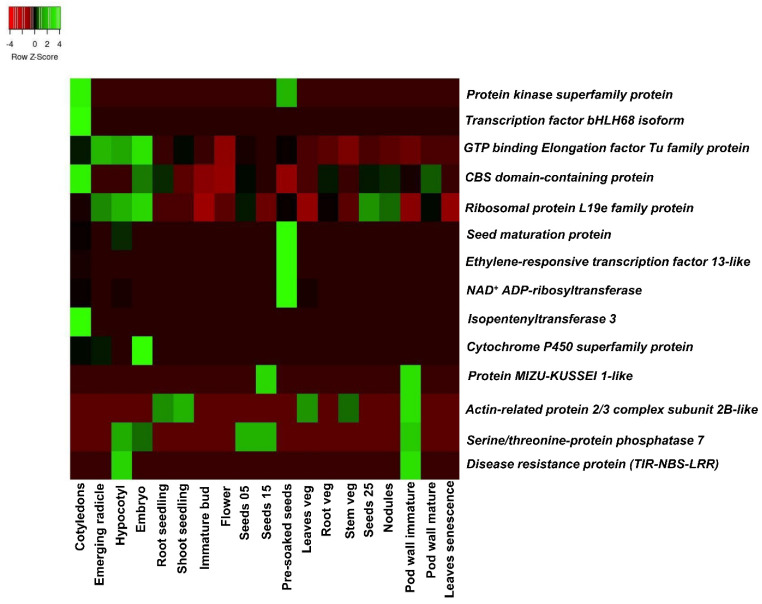
Tissue-specific expression of candidate genes across 19 different tissues detected underlying major QTLs for HSW and SP.

The major QTLs for HSW and SP were further investigated for putatively governing genomic regions because of the comparatively higher phenotypic variance than other traits (Table 4). A total of ten candidate genes were identified in the major QTL region (S16_2332048-S16_8231918) on the B06 chromosome for HSW, and four genes were identified in the major QTL region of S12_42838843-S12_73270208 on B02 for SP (Table 6). These genes had functional annotation directly or indirectly related to HSW and SP. The gene expression atlas (AhGEA) of *A. hypogaea* ssp. *fastigiata* was used to identify the tissue-specific expression of these genes [46]. Based on functional annotations, the genes and transcription factor located in the major QTL region for HSW and SP were involved in the signaling cascade, biotic and abiotic stress mechanism, and flavonoid synthesis, e.g., the seed maturation protein (*Araip.GWR7V*) and serine-threonine phosphatase-encoding gene (*Araip.DH675*) were highly expressed on seeds and pod walls (Figure 4), suggesting their role in seed development, and they can be positively correlated with the increase in HSW. Similarly, TIR-NBS-LRR (*Araip.6MG4Z*), the protein kinase superfamily (*Araip.49T7Y*), the bHLH transcription factor (*Araip.5E3CZ*), isopentyl-transferase (Araip.UY42T), the CBS-domain-containing protein (*Araip.CXF88*), the ethylene-responsive transcription factor (*Araip.LE5CL*), and the cytochrome P450 superfamily gene (*Araip.WM0UU*) were found in the genomic region and showed tissue-specific expression (Figure 4). 

## 4. Discussion

On a global scale, the adverse effects of climate change on crop productivity are evident, as they amplify various biotic and abiotic stresses, highlighting the urgency to improve existing cultivars. Consequently, strategies to exploit genetic variation become essential for peanut breeding as resistance against biotic and abiotic stresses directly impact peanut production. The utilization of genomics helps in targeting complex traits such as yield for improvement and utilizing novel alleles from wild species. Key traits like pod and seed weight are directly reflective of yield and have been widely studied in peanuts and other crops [48,49,50,51]. Initially, five SSR markers associated with pod- and kernel-related traits were identified through bulk segregant analysis [16]. Subsequently, in the F_2_ population (Zhonghua 10 × ICG12625), twenty-four QTLs (PVE- 1.69–18.70%) for HPW, HSW, SP, main stem height, pod length, seed length, pod length, and pod width were identified [52]. Additionally, for shelling percentage, 25 QTLs were identified in the RIL population (Yuanza 9102 × Xuzhou 68-4) [53]. These findings not only offer insight into gene discoveries but also help in the identification of functional markers for breeding.

In this study, a wide range of yield- and quality-related traits was observed for the RILs evaluated in two different environments, confirming the existence of genetic variability for different yield- and quality-related traits in peanuts [27,52,53]. The population exhibited significant variation among the lines for haulm yield, oil content, protein content, linoleic acid, and oleic acid, indicating high variability among the tested RILs for the respective traits. A G × E interaction study is typically conducted to assess the adaptability and stability of lines or cultivars across different environments for quantitative traits. The higher the environmental variance, the higher the differential expression of lines across the environments. The environments used in the current study showed the inconsistency of RILs for some traits. Information on G × E interaction for yield- and quality-related traits is essential to develop effective selection strategies to improve yield in variable environments. The significant G × E interaction for pod yield, HPW, HSW, SP, and SCMR in the present study indicates inconsistent lines across environments, which was reported earlier [54], emphasizing the importance of examining the lines in different environments. The performance of RILs varied significantly between environments due to large G × E interactions for pod yield, HPW, HSW, SP, and SCMR.

Yield-attributing traits have a complex interaction pattern, elucidated through the information of quantitative trait loci (QTL). These QTLs influence traits through cumulative effects. Moreover, epistatic interactions among minor loci affect multiple traits and must be incorporated along with QTLs’ introgression in the breeding program. Efforts were made to identify QTLs/genes associated with yield- and quality-related traits, followed by developing the lines by introgressing the selected genomic regions such as biotic and abiotic stresses through MAS [55,56,57]. Using a high-density 58K “Axiom_*Arachis*” array [58] and RILs from a cross between TAG 24 and ICGV 86031, 1205 SNP loci spanning 2598.3 cM were mapped, with an average marker distance of 2.2 cM [38]. The current work developed a linkage map comprising 1323 SNP loci, covering a total map length of 2003.13 cM, with an average marker distance of 1.89 cM between adjacent loci using the RIL mapping population. QTL analysis revealed that, except for four QTLs (two for HSW and two for SP), the phenotypic variance explained by the remaining QTL was <10%. It showed the complex nature of inheritance in peanuts. These observations support the previous report of multiple QTLs with minor effects associated with flowering date and maturity period in peanuts [27]. Similarly, an RIL population (JH5 × M130) was used to construct a genetic map using 3130 markers, detecting QTLs for 100-pod weight and 100-seed weight on chromosomes A03, A04, A08, B04, B05, B06, and B08 of peanuts. A new genomic region of 0.36 Mb on chromosome A08 was detected as a hotspot, including 18 candidate genes [48]. Moreover, the genomic regions for 100-seed weight and shelling percentage were also identified using the RIL population (Chico × ICGV 02251). QTL analysis identified three consistent QTLs on chromosomes A05, A08, and B10, whereas seven QTLs were found on chromosomes A01, A02, A04, A10, B05, B06, and B09 for 100-seed weight [59]. In a study utilizing an RIL population derived from the cross between JH6 and KX01-6, two stable QTLs (*qHYF_A08* and *qHYF_B06*) were identified across six different environments. The QTL *qHYF_A08* showed a predominant association with variations in shelling percentage and 100-pod weight, exhibiting PVE values ranging from 5.78% to 23.20%. Conversely, *qHYF_B06* was primarily linked to variations in 100-pod weight and 100-seed weight, with PVE values ranging from 13.38% to 31.29% [60].

The phenomenon of consistent QTLs detected under different environments with significant G × E interaction was reported in peanuts [61,62]. Two major QTLs mapped for HSW and two for SP were on B06 and B02, respectively. Thus, chromosomes B02 and B06 harbored important regions for SP and HSW, respectively. Similarly, the major and consistent QTL, *cqSPB02,* was identified on chromosome B02 for shelling percentage, with phenotypic variance explained being 10.47–17.01% across four different environments in the RIL population (Yuanza 9102 × Xuzhou 68-4) [53]. Moreover, three major QTLs (*q100SW16a*, *q100SW16a,* and *q100SW16a*) were found on chromosome B06 for 100-seed weight having PVE of 29.81–35.39% across four different seasons [63], indicating stable genetic effects independent of environments. The primary influence of a QTL cannot be solely attributed to the genetic background; in certain cases, it could be influenced by environmental factors or a combination of both. One notable QTL for HSW was located on chromosome B05, as well as two significant QTLs for shelling percentage which were found on chromosomes B06 and B10, which demonstrated substantial additive effects influenced by the environment [59]. 

The common QTL regions governing different traits suggest the relationship between these traits, pleiotropy effects, and/or tightly linked genes [5]. The genomic region associated with HSW between the marker interval of S16_2332048 and S16_8231918 on chromosome B06 harbored ten genes. These genes encoded the protein kinase superfamily protein, transcription factor *bHLH68*, the *GTP binding elongation factor Tu family protein*, the *CBS domain-containing protein*, the *ribosomal protein L19e family protein*, the *seed maturation protein*, the *ethylene-responsive transcription factor*, *NAD + ADP-ribosyltransferase*, *isopentenyltransferase* and the *cytochrome P450 superfamily protein*. Similarly, the genomic region associated with SP (S12_42838843-S12_73270208) on chromosome B02 harbored four genes. These genes encoded protein *MIZU-KUSSEI 1*, the *actin-related protein*, *serine/threonine-protein phosphatase* and the *disease resistance protein* (*TIR-NBS-LRR*). Nine of the fourteen genes had annotations directly or indirectly related to yield-related traits. 

The disease resistance protein coding gene (*Araip.6MG4Z*) was highly expressed in seeds and detected in the major genomic region of shelling percentage (Figure 4). This reveals that the disease resistance protein, *TIR-NBS-LRR,* may be indirectly involved in pod and seed development in addition to providing resistance to the plant against disease infestation. This gene is a part of receptor-like kinase gene family, known for salt tolerance and low temperature resistance induced by ABA [38]. The *serine-threonine phosphatase*-encoding gene (*Araip.DH675*) was highly expressed on pod walls and found to be associated with signal transduction for cell division and differentiation. The *serine-threonine phosphatase protein*-encoding gene was also reported in the B02 chromosome associated with shelling percentage in peanuts [61]. In rice, the *serine-threonine phosphatase* gene contains the Kelch motif, which determines the larger grain size and thus contributes to yield increment [64]. Similarly, the *serine-threonine phosphatase* gene also identified in the major QTL region was associated with pod length in soybean [65]. In maize, the role of the *serine/threonine protein kinase*-encoding gene *KNR6* was reported for ear length, and the overexpression of this genomic region resulted in significantly increased yield [66]. 

The gene protein kinase superfamily (*Araip.49T7Y*) is expressed in cotyledons and seeds. A kinase protein such as mitogen-activated protein kinase (MPK3) regulates the mitotic activities in the integumental cells through phosphorylation. These proteins may be involved in pod and seed development through protein–protein interactions [67]. The role of *calcium dependent protein kinase* (*CDPK*) was evaluated in developing peanut pods [68]. The higher expression of CDPK in early pod development might suggest that the absorption of Ca^2+^ occurs directly through the epidermal layer of pods in addition to via the xylem route. This argument was supported by the transcriptional upregulation of CDPK only in the development of seeds in Ca^2+^-deficient zones [68]. The *seed maturation protein coding* gene (*Araip.GWR7V*) is expressed higher in seed tissues than other vegetative tissues, indicating its role in seed maturation and development. The upregulation of the seed maturation protein [69] in seed tissues is consistent with the high activity of protein synthesis in seeds. Genes related to seed maturation, such as those involved in the seed storage protein and the accumulation of lipids, are usually regulated by the interaction of cis-acting elements in the promoter region and transcriptional regulators [70]. These regulatory networks promote the accumulation of seed storage reserves and thus lead to an increase in seed weight. Further, the expression pattern of the *Araip.GWR7V* gene was preferentially higher in seeds, which indicated that the promoter region of *Araip.GWR7V* can function in a seed-specific manner [71]. The *isopentyltransferase* (*IPT*) gene (*Araip.UY42T*) is one of the critical enzymes involved in cytokinin biosynthesis. The *IPT*-expressing peanut plant was identified with higher biomass in a dryland condition in the field [72]. This significant positive correlation of the higher yield of IPT-expressing plants with an increase in photosynthesis indicated the role of the cytokinin-mediated regulation of photosynthesis in transgenic plants. The differential expression pattern of the *IPT* gene showed that the regulatory function of the IPT gene in cytokinesis biosynthesis was one of the prime factors for determining pod size in peanuts [73]. The ethylene-responsive transcription factor (*Araip.LE5CL*) is highly expressed in seeds, and previously, it was reported in associated genomic regions of haulm weight [37]. The important role of ethylene-responsive transcription factors in the early development of peanut pods has also been identified [74]. Generally, Ca^2+^ ions promote pod maturation and development; however, the downregulation of genes encoding the ethylene-responsive transcription factor was reported in the presence of Ca^2+^ [75]. This indicated that ethylene-responsive transcription factors have a negative correlation with pod formation and development. Moreover, the ethylene-responsive element-binding factor family has a large number of transcription factors which are involved in abiotic and biotic stresses in plants [76,77]. The ethylene-responsive transcription factor superfamily genes, namely *GmAP2-1*, *GmAP2-2*, *GmAP2-3*, *GmAP2-4*, *GmAP2-5*, *GmAP2-6,* and *GmAP2-7,* had important roles in the regulation of seed length and seed width in overexpressed transgenic lines of Arabidopsis [78]. The members of the family of the *cytochrome P450 protein-encoding* gene (*Araip.WM0UU*) are involved in brassinosteroid biosynthesis. The *CYP72C1* gene (a *cytochrome P450 monooxygenase family*) regulates cell elongation and therefore results in short petioles and shortened seeds along the longitudinal axis [79]. This study supports that members of the *cytochrome P450* gene affect the seed size and its elongation by regulating the brassinosteroid level [79]. The *CBS-domain-containing protein* (*Araip.CXF88*) was expressed at relatively higher levels in cotyledons than in other tissues (Figure 4). Similarly, the expression of the CBS-domain-containing protein in cotyledons and floral tissues in addition to anthers was reported in the *proCBSX1:GUS*-expressing transgenic line of *Arabidopsis* [80]. The overexpression of the *CBS-domain-containing protein* was reported to increase the soybean’s low nitrogen stress tolerance [81]. The *bHLH transcription factor* (*Araip.5E3CZ*) is specifically expressed only in cotyledon tissues, which may explain its role in seed development (Figure 4). In addition, it also participates in other developmental processes such as the proper growth of axillary meristems, root hair, and anthers [82]. The higher expression of *bHLH* TFs in peanut seed tissues signifies its importance in seed development and maturation [38,83] and its pleiotropic role in plant growth and development as well as in stress responses [84,85]. Similarly, a bHLH transcription factor (TaPGS1) was specifically overexpressed in wheat and rice lines, which resulted in increased grain weight [86]. In addition, a yeast one-hybrid assay showed that the overexpression of the bHLH transcription factor AhbHLH121 resulted in the increased activity of antioxidant enzymes under stress by facilitating the expression of the genes for peroxidase, catalase, and superoxide dismutase in peanuts [87].

In addition, epistatic effects (the interaction of different loci in a population) play a significant role in determining trait expression [88,89]. A total of 91 pairs of QTL interactions were detected for all traits, suggesting that apart from environmental effects, epistatic QTLs also play a non-additive role in the inheritance of these traits. Such results are not surprising given that epistasis is more important for traits governed by several QTLs with small effects than for those governed by a few large major QTLs [90]. Epistatic QTLs affecting more than one trait were also reported in peanuts for pod number per plant [91]. Likewise, a total of 73 pairs of epistatic interactions involving 92 loci were discovered for pod length, pod width, length–width ratio, pod roundness, beak degree, and constriction degree. These interactions collectively accounted for phenotypic variations ranging from 0.94% to 6.45% [92]. 

## 5. Conclusions

A SNP-based genetic map consisting of 1323 loci and spanning 2003.12 cM was constructed. A total of 17 QTLs were detected for nine yield- and quality-related traits. Out of four, two each for HSW and SP were major QTLs. Notably, the major QTLs for HSW, detected on the B06 chromosome, exhibited a PVE of up to 14.65%. Similarly, major QTLs for SP, identified on the B02 chromosome, had a PVE of up to 11.65%. These major QTLs harbored genes and TFs which could affect the yield. The study demonstrated the significant impact of both individual- and multiple-effect epistatic QTLs in influencing the phenotype of various traits by interacting with genomic regions on different chromosomes. Within the identified major QTL regions, a total of ten candidate genes were pinpointed for HSW and four candidate genes for SP. Further investigation into the genomic region associated with the seed maturation protein is recommended. This exploration could aid in the development of markers important for enhancing peanut breeding efforts geared towards increasing productivity.

## Figures and Tables

**Table 1:** Summary of pooled ANOVA for yield- and quality-related traits in Valencia-C × JUG-03 RIL population.

Trait/Source of Variation	Mean Sum of Squares				
	Environment	Replication	Genotypes	G × E	Pooled Error
Degrees of freedom	1	1	143	143	286
Pod yield (kg ha^−1^)	9,047,419.28 *	207,621.32	40,307.69	61,125.05 ***	27,562.89
Haulm yield (kg ha^−1^)	38,793,685.30 *	227,604.46	56,512.90 *	55,332.65	36,921.52
HPW (g)	680.68	1676.18	120.94	107.35 ***	66.61
HSW (g)	1040.53	665.70	31.87	31.657 ***	17.71
SP	7696.37	2031.01	53.69	66.38 ***	39.92
SCMR	1948.92	439.82	46.98	44.46 ***	24.82
Oil content (%)	470.49	1130.28	31.43 ***	12.79	9.51
Protein content (%)	2.08	194.32	5.22 ***	2.68	1.48
Linoleic acid (%)	23.66	77.20	99.13 ***	26.02	17.11
Oleic acid (%)	526.32	867.12	137.77 ***	30.62	18.65

* and ***: significant at 5 and 0.1 percent level of probability, respectively; G × E: genotype × environment interaction; HPW: 100-pod weight (g); HSW: 100-seed weight (g); SP: shelling percentage; SCMR: SPAD chlorophyll meter reading.

**Table 2:** Mean, range, and variability components in individuals and pooled across two environments for yield- and quality-related traits in Valencia-C × JUG-03 RILs.

Traits	Environment	Parental Means		CD (5%)	Recombinant Inbred Lines				
		JUG-03	Valencia-C		RIL	Range	PCV	GCV	H^2^
					Mean				
Pod yield	E1	580.00	890.00	266.72	675.24	580–960	25.73	16.20	39.67
	E2	299.16	690.75	371.78	420.84	121.75–725.16	51.37	25.29	24.25
Haulm yield	E1	465.07	297.90	171.44	434.71	229.67–608.5	24.98	15.03	36.20
	E2	991.50	654.5	509.05	953.75	646.5–1418	9.22	28.53	10.45
HPW	E1	66.61	94.62	22.09	78.09	61.62–102.06	16.37	7.95	23.57
	E2	74.50	82.00	6.14	75.93	73–86	5.50	3.67	44.56
HSW	E1	30.00	39.50	11.69	34.48	28–44	20.23	10.74	28.15
	E2	30.05	32.60	1.33	31.79	29.9–33.2	2.78	1.79	41.58
SP	E1	47.00	58.49	14.63	53.07	45.5–63.11	15.28	6.23	16.61
	E2	52.11	66.85	9.79	60.41	50.87–71.5	9.54	4.86	26.03
SCMR	E1	42.50	55.35	11.44	45.32	37.5–59.2	15.39	8.59	31.13
	E2	37.60	46.15	7.94	41.64	34.9–48.35	11.23	5.75	26.24
Oil content	E1	46.23	42.47	7.62	44.26	38.41–48.94	10.11	5.14	25.87
	E2	49.28	40.41	5.73	46.08	39.21–55.33	8.04	5.00	38.66
Protein content	E1	30.50	25.98	2.52	28.91	23.03–31	5.04	2.43	23.21
	E2	30.22	25.75	2.27	28.78	23.03–30.22	5.08	3.13	38.08
Linoleic acid	E1	24.47	33.10	9.46	27.91	17.01–36.23	20.35	10.96	29.02
	E2	22.55	30.73	9.94	27.84	12.05–34.59	24.74	16.9	46.65
Oleic acid	E1	42.21	52.92	11.98	49.15	38.51–60.86	14.3	7.24	25.65
	E2	44.14	50.32	12.9	47.24	35.59–60.15	17.39	10.56	36.92

CD: critical difference at 5% level of significance; H^2^: broad sense heritability; PCV: phenotypic coefficient of variance; GCV: genotypic coefficient of variance; HPW: 100-pod weight (g); HSW: 100-seed weight (g); SP: shelling percentage; SCMR: SPAD chlorophyll meter reading.

**Table 3:** Summary of genetic map constructed using single-nucleotide polymorphism (SNP) markers.

Chromosome	Mapped Loci	Map Distance (cM)	Inter-Marker Distance (cM)	Map Density (loci/cM)
A01	58	99.50	1.72	0.58
A02	42	134.70	3.21	0.31
A03	82	142.69	1.74	0.57
A04	61	88.70	1.45	0.69
A05	67	129.80	1.94	0.52
A06	42	75.30	1.79	0.56
A07	22	55.00	2.50	0.40
A08	45	167.12	3.71	0.27
A09	47	87.30	1.86	0.54
A10	92	99.36	1.08	0.93
B01	51	112.02	2.20	0.46
B02	99	137.54	1.39	0.72
B03	89	101.21	1.14	0.88
B04	61	71.10	1.17	0.86
B05	124	74.85	0.60	1.66
B06	91	126.80	1.39	0.72
B07	98	119.96	1.22	0.82
B08	21	74.00	3.52	0.28
B09	20	72.50	3.63	0.28
B10	111	70.28	0.63	1.58
Grand Total	1323	2003.13	1.89	

**Table 4:** List of the QTLs for different traits across two environments, identified by QTL Cartographer.

QTLs	Traits	Env.	Chr.	Pos. (cM)	Marker Interval	LOD	PVE (%)	Additive Effect
*qPODYLD18E1*	Pod yield	E1	B08	28.17	S18_51822496-S18_134813874	4.88	6.87	−155.85
*qPODYLD12.2E2*	Pod yield	E2	B02	99.71	S12_93246314-S12_100066236	4.6	6.27	−65.62
*qPODYLD12.3E2*	Pod yield	E2	B02	106.01	S12_110782372-S12_112245066	4.78	6.53	−65.04
*qHAULMYLD18E1*	Haulm yield	E1	B08	28.61	S18_51822496-S18_134813874	4.34	6.09	−64.16
*qHAULMYLD6E1*	Haulm yield	E1	A06	29.16	S6_1562649-S6_1630272	3.64	9.55	149.88
*qHSW12E1*	HSW	E1	B02	57.21	S12_42838843-S12_73270208	4.06	5.89	9.39
*qHSW16E1*	HSW	E1	B06	120.57	S16_2332048-S16_8231918	6.65	14.65	−5.38
*qHSW16E2*	HSW	E2	B06	120.55	S16_2332048-S16_8231918	6.11	13.87	−3.31
*qSP12E1*	SP	E1	B02	57.23	S12_42838843-S12_73270208	4.36	10.98	13.95
*qSP12E2*	SP	E2	B02	57.21	S12_42838843-S12_73270208	4.43	11.65	5.44
*qSCMR17E2*	SCMR	E2	B07	22.21	S17_32203546-S17_66864943	4.27	5.9	−6.28
*qOIL12E1*	Oil content	E1	B02	57.21	S12_42838843-S12_73270208	3.05	4.49	10.65
*qOIL12E2*	Oil content	E2	B02	57.23	S12_42838843-S12_73270208	3.02	4.46	10.67
*qPROTEIN12E2*	Protein content	E2	B02	57.21	S12_42838843-S12_73270208	3.15	4.66	6.79
*qLINOLEIC12E2*	Linoleic acid	E2	B02	64.11	S12_36421620-S12_118023921	3.43	5.15	−3.41
*qOLEIC17E1*	Oleic acid	E1	B07	22.21	S17_32203546-S17_66864943	5.76	8.09	−9.09
*qOLEIC17E2*	Oleic acid	E2	B07	22.21	S17_32203546-S17_66864943	6.14	8.5	−9.16

Env.: environment; Pos.: position; Chr.: chromosome; LOD: logarithm of odds; PVE: phenotypic variance explained (%); HPW: 100-pod weight (g); HSW: 100-seed weight (g); SP: shelling percentage; SCMR: SPAD chlorophyll meter reading.

**Table 5:** Summary of epistatic QTLs (E-QTLs) for yield- and quality-related traits identified by ICIMapping.

Traits	No. of QTLs	LOD Range	PVE %
Pod yield	9	3.08–4.18	24.96–38.48
Haulm yield	15	3.00–5.97	11.86–54.84
HSW	4	3.04–4.17	9.31–20.48
SP	14	3.00–5.72	11.72–72.61
SCMR	8	3.00–4.10	11.85–22.85
Oil content	7	3.04–4.77	11.63–28.23
Protein content	11	8.54–19.99	48.31–61.59
Linoleic acid	2	3.02–3.30	18.75–26.73
Oleic acid	7	3.08–4.38	13.07–50.08

LOD: logarithm of odds; PVE: phenotypic variance explained (%); SP: shelling percentage; SCMR: SPAD chlorophyll meter reading; HSW: 100-seed weight.

**Table 6:** Fourteen putative genes identified in the genomic region associated with SP and HSW.

Traits	QTL Name	Gene Location	Gene Model	Nearest SNP (bp)	Functional Annotation
SP	*qSP12E1 and qSP12E2*	Araip.B02	*Araip.6MG4Z*	67,500,914	Disease resistance protein
			*Araip.DH675*	42,817,549	Serine/threonine-protein phosphatase
			*Araip.5HJ7H*	67,500,914	Protein MIZU-KUSSEI 1
			*Araip.7QC0C*	42,817,549	Actin-related protein
HSW	*qHSW16E1 and qHSW16E2*	Araip.B06	*Araip.49T7Y*	8,393,784	Protein kinase superfamily protein
			*Araip.5E3CZ*	1,562,649	Transcription factor bHLH68
			*Araip.CXF88*	271,015	CBS domain-containing protein
			*Araip.GWR7V*	2,818,988	Seed maturation protein
			*Araip.LE5CL*	1,562,649	Ethylene-responsive transcription factor
			*Araip.UY42T*	2,697,960	Isopentenyltransferase 3
			*Araip.WM0UU*	7,821,084	Cytochrome P450 superfamily protein
			*Araip.60J6J*	2,818,988	GTP binding elongation factor
			*Araip.CY9QC*	8,393,784	Ribosomal protein L19e family protein
			*Araip.SKT5W*	1,562,649	NAD^+^ ADP-ribosyltransferase

HSW: 100-seed weight; SP: shelling percentage.

## Data Availability

Data presented in the study are deposited in NCBI, accession number PRJNA1036023.

## Supplementary Materials

The following supporting information can be downloaded at: https://www.mdpi.com/article/10.3390/genes15020140/s1, Supplementary Table S1: ANOVA of RIL population for 10 traits in different environments; Supplementary Table S2: Combined analysis of variance for pooled data of RIL population; Supplementary Table S3: Threshold LOD determined by permutation run 1000 times for each trait separately; Supplementary Table S4: List of epistatic QTLs for yield- and quality-related traits across two environments, identified by ICIMapping.
